# Iron Nitrates Stimulate Phenolic Compound Production by *Melissa officinalis* L.

**DOI:** 10.3390/ijms27146211

**Published:** 2026-07-11

**Authors:** Dorota Adamczyk-Szabela, Wojciech M. Wolf, Dawid Krakowiak

**Affiliations:** Institute of General and Ecological Chemistry, Lodz University of Technology, Zeromskiego 116, 90-924 Lodz, Poland; wojciech.wolf@p.lodz.pl (W.M.W.); dawid.krakowiak@dokt.p.lodz.pl (D.K.)

**Keywords:** heavy metals, iron supplementation, photosynthesis, herbs, total phenolic compounds

## Abstract

Iron plays a key role in plant chemical processes and photosynthesis. The aim of this study was to investigate the effect of various iron concentrations on total phenolic compound levels and photosynthetic efficiency in *Melissa officinalis*. It is a very popular herb, widely cultivated worldwide for its medicinal properties. Herbs were grown under controlled laboratory conditions. Gas exchange parameters, chlorophyll index, and total content of phenolic compounds were determined. Additionally, iron, manganese, copper, and zinc concentrations in either soil or plant tissues were analyzed. The hypothesis that iron supplementation significantly affects photosynthesis and total phenolic compounds in lemon balm was confirmed by one-way analysis of variance. In particular, the beneficial biofortification at 100 and 200 µg/g iron doses generates a moderate increase in phenolic compounds and simultaneously does not hamper plant growth and photosynthetic efficiency. However, iron doses above 500 µg/g induce plant stress and trigger defense mechanisms that significantly increase phenolic compound production. This is accompanied by a decrease in biomass and photosynthetic efficiency.

## 1. Introduction

*Melissa officinalis* L. (lemon balm) is a perennial herb from the mint family, extensively cultivated worldwide and in Poland [1]. The chemical compounds present in the plant are effective in treating nervous agitation, stress, chronic fatigue syndrome, anxiety, vegetative neuroses, and digestive problems [2]. Biologically active compounds contained in lemon balm leaves give it antibacterial, antifungal, and antiviral properties [3,4]. Furthermore, the herb contains phenolic compounds, which are responsible for its antioxidant properties.

In stress conditions induced by abiotic factors, including heavy metals, plants moderate their response by increasing the production of phenolic compounds. Phenolic compounds reduce oxidative stress not only by directly scavenging free radicals but also by inhibiting the activity of enzymes responsible for their formation and lipid peroxidation. Specifically, they inhibit NADPH and xanthine oxidases which generate reactive oxygen species, as well as lipoxygenase and cyclooxygenase [5]. The latter two enzymes participate in lipid peroxidation. Phenolic compounds also increase the activity of important antioxidant enzymes, i.e., superoxide dismutase and catalase [6]. Stress in plants can be induced not only by an excess of metals in the environment but also by a deficiency of nutrients. Iron can serve as an example, as it plays a key role in plant development.

Approximately 40% of the world’s cultivated areas are affected by soil iron deficiency [7], which negatively impacts crop quality. While the iron total content in soils fluctuates between 0.5 and 5% [8] the soluble iron levels are usually exceptionally low [4,9]. Following Korzeniowska et al. [10], iron content below 700 mg/kg of dry soil is considered as low while 700–3800 mg/kg and 3800 mg/kg concentrations are referred to as medium and high levels, respectively. Iron solubility in soil is dependent on many reactions, particularly hydrolysis and the content of complex compounds [11]. Furthermore, iron mobility in soil is largely controlled by the solubility of amorphous hydrated Fe^3+^ and Fe^2+^ oxides and the formation of other iron compounds [12]. Iron participates in numerous processes [13], including protein synthesis, DNA replication, photosynthesis, and plant respiration [3,14,15,16,17]. Iron content in plants is usually in the range from 50 to 200 mg/kg of dry weight and depends on several factors such as soil pH, organic matter, moisture, temperature, and interactions with other elements [18,19]. Iron uptake by plants is metabolically controlled, although it can be absorbed as Fe^3+^, Fe^2+^ or Fe-chelates [17]. Separation of chelated Fe prior to absorption appears to require reduction of Fe^3+^ to Fe^2+^ at the root surface [20,21,22]. In xylem exudates, iron occurs in an unchelated form, but its transport is primarily mediated by citrate chelates [23,24,25,26]. Iron deficiency causes metabolic disturbances in plants, leading to decreased chlorophyll index and reduced photosynthesis [27,28]. The primary sign of iron deficiency in plants is chlorosis, which occurs due to the degradation of chlorophyll [29]. In agricultural practice, iron deficiencies in plants are not related to low levels of iron in the soil, but rather to its limited availability to plants due to soil conditions. This is often caused by intensive liming, overfertilization, or periodic flooding. Iron fertilization requires appropriate soil conditioning to ensure that the element is absorbed most effectively. It is essential to select the appropriate dose to suit the individual needs of the plant. Furthermore, iron cations play a crucial role in regulating the mobility of macronutrients and trace elements in plants. During soil overfertilization, high iron concentrations can be potentially toxic to plants and lead to the formation of reactive oxygen species. The available literature primarily focuses on soil iron deficiency, which directly stimulates the biosynthesis and secretion of phenolic compounds in plants [30,31]. There are only a few reports on iron fertilization of plants. However, there is an astonishing lack of data on herbs supplemented with different doses of iron.

The aim of this study was to investigate the response of lemon balm to various levels of Fe^3+^ supplementation. We focused on the determination of a plant-safe dose that would enrich lemon balm with iron and additionally increase the level of phenolic compounds in the plant’s tissues. This should enhance the pharmaceutical value of this herb.

In Central Europe’s climate zone, supplementation with phenolic compounds is often necessary for people consuming a diet low in these compounds. According to the available literature, consuming foods rich in phenolic compounds reduces the likelihood of cardiovascular disease, heart disease, and cancer [32,33,34]. Various preparations containing freeze-dried extracts, such as those from bergamot fruit, with standardized concentrations of phenolic compounds are available on the market [35,36].

Nevertheless, there is a clear need to expand the range of plants from which phenolic extracts can be successfully obtained. It is well known that lemon balm extracts are considered safe, and allergic reactions induced by them are extremely rare [37,38].

It is generally known that phenolic acids can bind Fe^3+^ and Fe^2+^ ions, modifying their solubility and mobility [39]. Not all plant species exhibit such a clear, measurable response, making lemon balm an excellent experimental model. However, further research on the antioxidant activity of plants is clearly needed. This is particularly due to the rising consumer concerns over novel antioxidants which are likely to enter the market [40].

Our preliminary results indicated that low iron supplementation enhanced photosynthesis by increasing chlorophyll production in herbs. The opposite effect was observed with high iron doses. In this work, we extend this observation over lemon balm cultivated in several carefully controlled conditions.

## 2. Results

### 2.1. Analysis of Soil Used for Plant Cultivation

The analysis of untreated soil showed that it belongs to acidic, organic soils [41,42]. According to international standards, the soil was not contaminated with iron, manganese, copper or zinc [43,44] (Table 1).

### 2.2. Lemon Balm Plant Analysis

#### 2.2.1. The Effect of Iron Supplementation on Heavy Metal Contents in Plants

**Figure 1:**
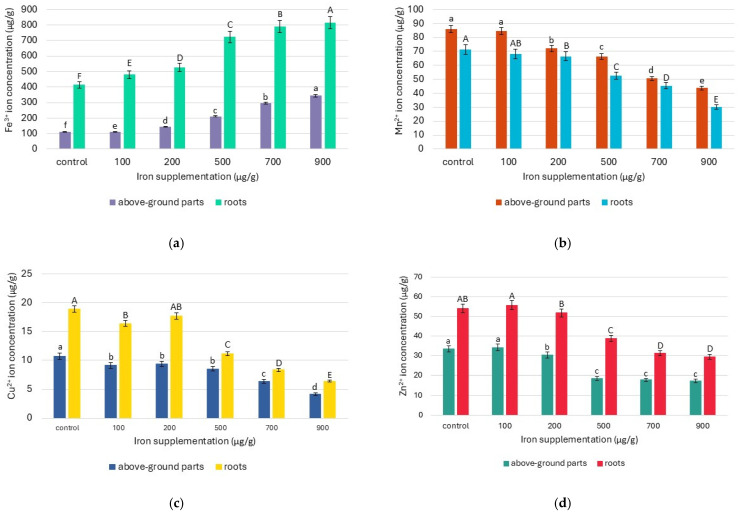
Iron (**a**), manganese (**b**), copper (**c**) and zinc (**d**) ion concentration in above-ground parts and roots of herbs cultivated in soil under iron supplementation. The number of samples *n* = 5. Within each plant part, values associated with different letters are significantly different according to Tukey’s HSD test at *p* = 0.05.

Metal contents in the above-ground parts and roots of lemon balm are shown in Figure 1. Lemon balm grown in soil without any additives (control) accumulates iron, copper and zinc in the roots and manganese in the above-ground parts. The null hypothesis was whether iron application affects the migration of metals from soil to plant (Table 2). In all tested plants, the increase in iron content hindered the uptake of all the determined metals from the soil and their transport to the roots and above-ground parts.

Metal uptake by plants can be estimated using bioaccumulation factors (BAFs), translocation factors (TFs) and transfer coefficients (TCs) (Table 3). BAF is defined as the ratio of the metal concentration in the above-ground parts of the plant to its content in the soil, TF is the ratio of the metal concentrations in the above-ground parts and roots of the plant, respectively, while TC is the ratio of metal content in roots to its content in the soil [45].

#### 2.2.2. The Effect of Iron Supplementation on Plant Growth and Photosynthesis

**Figure 2:**
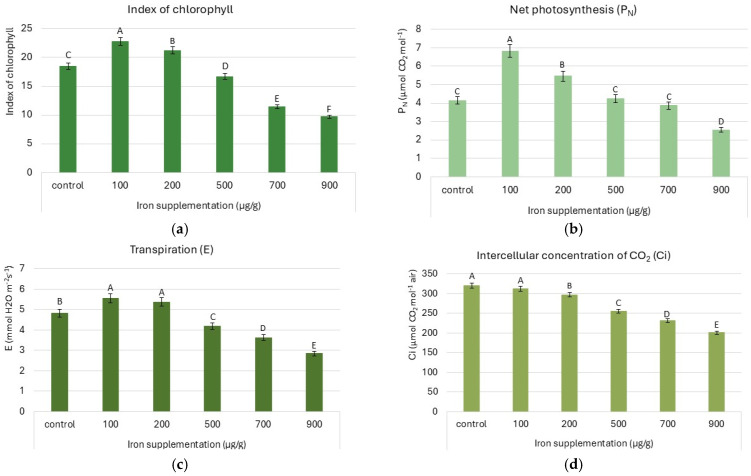

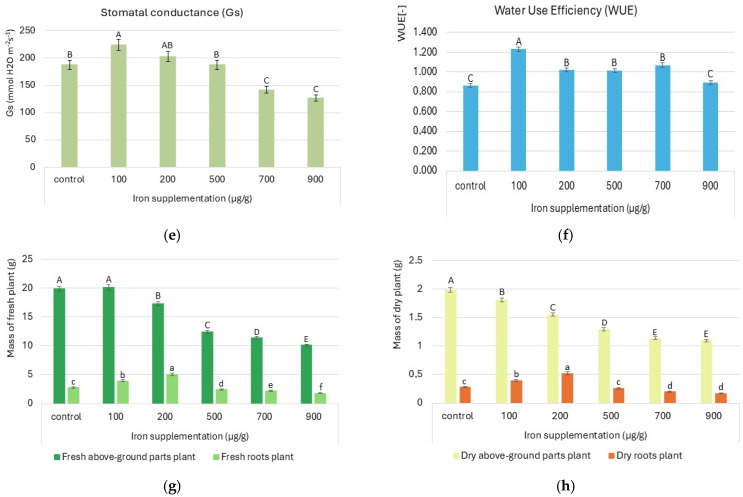
Index of chlorophyll (**a**), net photosynthesis (**b**), transpiration (**c**), intercellular concentration of CO_2_ (**d**), stomatal conductance (**e**) water use efficiency (**f**), fresh plant mass (**g**) and dry plant mass (**h**) determined for lemon balm with relevant standard deviations (*n* = 5). Within each plant part, values associated with different letters are significantly different according to Tukey’s HSD test at *p* = 0.05.

The gas exchange parameters, which illustrate the photosynthesis efficiency, i.e., index of chlorophyll, net photosynthesis (P_N_), transpiration (E), stomatal conductance (G_S_), intercellular CO_2_ (Ci) and biomass of above-ground parts of plants, are shown in Figure 2. Low doses (100 and 200 µg/g) of iron increased photosynthesis yield substantially. Supplementations 500 and 700 µg/g stabilized P_N_ at the control level while the largest iron dose (900 µg/g) drove it to the lowest value observed. This situation is reflected by the chlorophyll index. The chlorophyll index in herbs treated with 100 and 200 µg/g Fe was significantly higher than control, as shown in Figure 2. However, higher doses of iron (500; 700; 900 µg/g) caused a gradual decrease in the chlorophyll index (10%; 38%; 48% respectively) in plants. Iron excess did not affect the intercellular CO_2_ significantly. A gradual decrease in C_i_ content was observed along the increasing concentration of the supplemented iron dose. Iron doses of 100 and 200 µg/g increased transpiration and stomatal conductance. However, supplementation with higher Fe^3+^ doses caused a significant decrease in transpiration and stomatal conductance compared to control. Plants control iron uptake by regulating water transport through stomatal opening. This affects transpiration, which lags behind net photosynthesis and reduces the intercellular CO_2_ concentration in the plant samples for which the highest photosynthetic efficiency was observed. Low doses enhanced the photosynthetic process while iron supplementation above 100 µg/g significantly inhibited lemon balm mass (Figure 2). To assess the effect of iron supplementation on photosynthesis, one-way ANOVA analysis was used at the probability level of *p* = 0.05 (Table 4).

#### 2.2.3. The Effect of Iron Supplementation on the Total Phenolic Compounds

**Figure 3:**
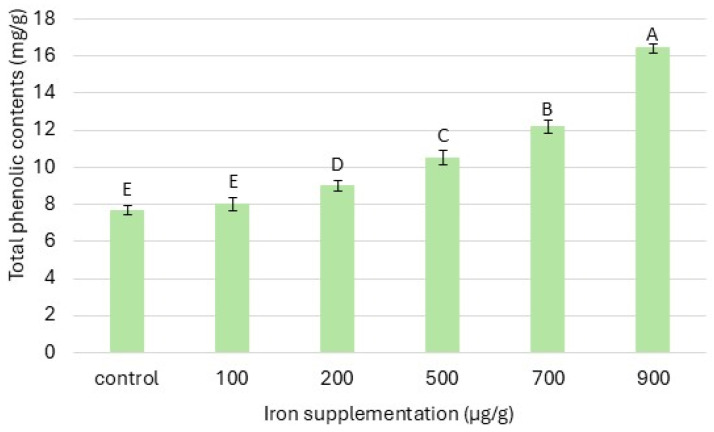
The total phenolic contents in above-ground parts of lemon balm as determined for control and iron supplementation accompanied by relevant standard deviations (*n* = 5). Within each plant part, values associated with different letters are significantly different according to Tukey’s HSD test at *p* = 0.05.

The levels of total phenolic compounds (TPCs), which were determined by the Folin–Ciocalteu procedure, are summarized in Figure 3. Extracts of lemon balm were characterized by a diversified content of total phenolic compounds. The highest amount was found in lemon balm supplemented with 900 µg/g iron (over 200%). Iron supplementation had a statistically significant effect on the total phenolic content in lemon balm (F = 240.063; *p* = 1.14 × 10^−19^; F_cryt_.(5,24) = 2.620; probability level *p* = 0.05).

## 3. Discussion

The availability of iron in the soil is often limited. In this respect, the application of fertilizers containing iron is the simplest way to deal with the symptoms of iron deficiency in plants. However, iron supplementation at high doses may be a stress factor which limits yield of cultivated herbs. A wide range of iron concentrations (100–900 µg/g) was used in our experiment. We aimed to encompass levels either close to the natural content of available iron in soil or elevated values that may cause physiological changes in plants.

Our results clearly demonstrate that high iron contents in the soil affect the manganese, zinc, and copper uptake and their subsequent migration within the lemon balm plant. The translocation factors calculated for plants grown in reference soil (control) are in the order Mn > Zn > Cu > Fe. Iron supplementations above 500 µg/g prompted a shift in the order between zinc and copper in the row. The transfer coefficients calculated for lemon balm cultivation in control soil are arranged in the following series: Cu > Zn > Mn > Fe. The addition of iron changed that order for all investigated metals. The largest decreases in TC values were recorded for copper after iron supplementation at a dose of 500 µg/g and for manganese at an iron concentration of 700 µg/g. Bioaccumulation factors for untreated soil followed the order Mn > Zn > Cu > Fe. Only zinc and copper changed their position within the series after 500 and 700 µg/g supplementations.

Iron supplementation decreased the BAF values for all investigated elements but did not affect their orders in respective series. According to data in the literature, Mn-Fe, Cu-Fe and Zn-Fe antagonisms are commonly known. This is due to the fact that zinc, manganese, copper and iron use similar protein transporters (ZIP, NRAMP and YSL) and compete for their active sites during the uptake [14,46]. This leads to nutrient imbalances and stunted plant growth. Furthermore, excess iron initiates ionic antagonism, in which Fe inhibits the uptake of Mn, Zn, and Cu through competitive interactions in the root zone. Furthermore, when plants absorb high doses of iron, they can secrete compounds in the root zone which alter the solubility of other metals and make them less available to the plant [47].

Iron is a key trace element for chlorophyll biosynthesis and the proper functioning of the photosynthetic apparatus. Iron deficiency leads to the inhibition of chlorophyll synthesis by blocking steps in the porphyrin pathway and results in leaf chlorosis [48]. Our results show that iron concentrations of 100 µg/g and 200 µg/g significantly increased chlorophyll content in lemon balm. Supplementations with applicable doses of iron increase the activity of biosynthetic enzymes and the level of chlorophyll precursors. Finally, they are likely to increase the chlorophyll content in plant tissues.

Furthermore, iron is a key cofactor of ferredoxin and plays a crucial role in electron transport during photosynthesis [49]. However, at high levels of supplementation, we observed a reduction in chlorophyll content in plants. Excess iron induced oxidative stress [50] by generating reactive oxygen species via the Fenton reaction [51], leading to chlorophyll degradation and chloroplast damage [52]. Furthermore, it affected the cellular ionic balance, disrupted chlorophyll biosynthesis and limited the synthesis of this pigment. In summary, either iron deficiency or iron excess reduced photosynthetic efficiency and prompted chlorotic changes in leaves [52].

Our results clearly showed an increase in net photosynthesis at iron concentrations of 100 and 200 µg/g. This was accompanied by a decrease in the intercellular CO_2_ which was likely due to the closure of stomata responsible for water transport regulation. The water use efficiency index and stomatal conductance approximately followed the patterns observed for net photosynthesis and transpiration. However, direct comparison of numerical data indicates that increased photosynthesis efficiency was accompanied by better use of water. This means that a higher proportion of water was directly used in photosynthesis. On the other hand, the reduction in water uptake was due to the stress effects stimulated by iron at high concentrations, which limited the growth of cultivated herbs. In our studies, a decrease in the fresh mass of the above-ground parts of the plant was observed after all iron applications. We therefore speculate that the latter affects the plant strategy and stimulated the investment of more energy into root system development. This hampers the growth of its green parts. There was a clear correlation between increased phenolic content and reduced plant weight, especially at iron doses above 500 µg/g. Plants treated with iron at a concentration of 900 µg/g showed the highest content of phenolic compounds. Two opposing processes therefore occurred. Beneficial biofortification with lower Fe doses generated a moderate increase in phenolic compounds and therefore did not negatively impact the plants’ growth or their photosynthetic efficiency. However, high Fe doses (above 500 µg/g Fe) induced plant stress and triggered defense mechanisms, which finally enhanced phenolic production. This was accompanied by a decrease in biomass and photosynthetic efficiency. We therefore reason that the limitation of photosynthesis at higher iron doses is due to the limited water availability as indicated by the C_i_ values. Under normal plant growth conditions, there is a balance between the production and detoxification of reactive oxygen species due to the metabolic processes in plants. However, under stress conditions, the free radical balance is disturbed, leading to the accumulation of reactive oxygen species. This results in the peroxidation of membrane lipids, which can initiate cell membrane destruction [53]. Plants may respond to this stress by increasing the activity of their antioxidant system. Antioxidant enzymes capture free radicals and prevent their formation [54]. Phenolic compounds are secondary metabolites with distinct free radical scavenging ability which increase plant tolerance to environmental stress. Plants adopt a plethora of diverse strategies to mitigate heavy metal toxicity. The first line of defense lies in the rhizosphere where root exudates interfere with metal ions and affect their uptake by plant tissues [55,56]. In particular, metal chelation hampers their entry into the symplast [57]. Root exudation of phenolic compounds is linked with metal concentration in the rhizosphere and differs substantially among diverse plant species [58,59]. Phenolic compounds play an essential role in the cultivation of lemon balm in iron-polluted soils. Our study shows that the total levels of phenolic compounds were systematically increased in all lemon balm cultivations supplemented with Fe^3+^. Unfortunately, biomass production consequently decreased. We would like to emphasize that herbal products with increased phenol content typically have a higher market value than the original herbs. Therefore, selecting an appropriate Fe^3+^ dose to increase phenolic content without compromising plant biomass is crucial. However, this requires further in-depth research and large-scale-cultivation field studies.

## 4. Materials and Methods

### 4.1. Soil Samples

Soil samples were collected from a farm located in Lagiewniki (51°51′ N, 19°28′ E; unfertilized and pesticide-free for two years from the beginning of the experiment; located away from traffic) according to PN-ISO 10381-4: 2007 [60] in August 2024. The soils were dried in an air-circulating room and passed through a 2 mm stainless steel sieve. Soil pH was measured in 1 mol/L KCl solution according to PN-ISO 10390:1997 by the potentiometric method [61]. Organic matter content in soil was determined by the gravimetric method [62,63]. The bioavailable forms of metals in soil were extracted by 0.5 mol/L HCl solution. A mixture of concentrated HNO_3_ (6 mL) and HCl (2 mL) was applied to soil (0.5 g) decomposed by minerals with the Anton Paar Multiwave 3000 closed system instrument (Graz, Austria). Metal contents were measured in soil samples by HR-CS FAAS with contraAA 300 spectrometer (Analytik Jena GmbH, Jena, Germany). Five replications were conducted for each sample.

### 4.2. Cultivation of Lemon Balm Material and Experimental Protocol

All herbs were cultivated under laboratory conditions by the pot method from March to July 2024. Lemon balm seeds (P.H. Legutko, Rawicz, Poland) were placed in 12 cm diameter plastic containers (0.3 L) filled with 500 g of soil. After germination, each container contained five plants. After one month of growth, lemon balm plants were treated with 5, 10, 25, 35, or 45 mL of 10 mg/mL Fe(NO_3_)_3_ solution, giving iron concentrations in the pots of 100, 200, 500, 700, and 900 µg/g, respectively. We used five parallel samples (i.e., five pots) in each cultivation series. The mean value was calculated from five independent pots. The cultivation was conducted in a greenhouse under controlled conditions: the temperature was 23 ± 2 °C and 16 ± 2 °C for day and night, respectively; the relative humidity was limited to 65–70%; the photosynthetic active radiation (PAR) during the 16 h photoperiod was restricted to 400 μmol m^−2^s^−1^. All plants were regularly watered with deionized water. The herbs were grown for four months. Then, the plants were cut and the above-ground parts and roots were separated, washed, dried at 45 °C to a constant weight and homogenized.

### 4.3. Morphological and Physiological Parameters of Plants

Measurements of photosynthesis parameters were made four months after sowing the seeds, just before cutting the plants. The index of chlorophyll was measured using the Konica Minolta SPAD-502Plus (Tokyo, Japan) chlorophyll meter. Net photosynthesis (P_N_), stomatal conductance (Gs), intercellular concentration of CO_2_ (Ci), and transpiration (E) were determined with the gas analyzer CIRAS-3 (Portable Photosynthesis System, Amesbury, MA, USA) (Table 5). All measurements were made five times on separate plants without damaging the plant material.

### 4.4. Determination of Heavy Metals in Herbs

Approximately 0.5 g of dried roots and 0.5 g aerial parts of the plants were weighed, 6 mL of concentrated HNO_3_ and 1 mL of concentrated HCl were added, and the whole was subjected to microwave mineralization in a closed system of the Anton Paar Multiwave 3000 apparatus (Graz, Austria). Metal contents were determined by HR-CS FAAS with a contraAA 300 spectrometer (Analytic Jena, Germany). Quality assurance and quality control (QA/QC) for metals in plant samples were estimated by determining metal contents in the certified reference material INCT-MPH-2, containing a mixture of selected Polish herbs [64] (Table S1).

### 4.5. Determination of Total Phenolic Content in Lemon Balm

The total phenolic content was determined by the Folin–Ciocalteu spectrophotometric method, with gallic acid applied as the standard [65]. Dried and ground herbs (1 g) were extracted with 40 mL of methanol (70%). The mixture was then centrifuged at 3000 rpm for 10 min and poured into plastic bottles. An amount of 0.1 mL of that extract, 6 mL of distilled water, and 0.5 mL of the Folin–Ciocalteu reagent were placed in a 10 mL volumetric flask and thoroughly mixed. After 3 min, 1.5 mL of saturated sodium carbonate solution was added and made up to the mark with distilled water. The solution was subsequently placed in a thermostat at 40 °C for 30 min. The absorbance was measured at a wavelength of 765 nm using a Specol 11 spectrophotometer (Carl Zeiss, Jena, Germany).

### 4.6. Statistical Analysis

Each determination was performed five times for independent samples. The normality of the data distribution was assessed using the Shapiro–Wilk test [66,67]. One-way analysis of variance (ANOVA) was used to identify significant differences in the phenolic content of iron-supplemented herbs. Tukey’s HSD post hoc test was used to assess statistically significant differences between the mean values within each variable.

Metal uptake by iron-treated herbs was assessed by one-way ANOVA at the 0.05 probability level (Table 2) and followed by post hoc Tukey’s test.

## 5. Conclusions

Low or moderate Fe(NO_3_)_3_ supplementation stimulated photosynthesis in lemon balm leaves through elevated chlorophyl synthesis. In particular, the increased photosynthesis efficiency prompted better use of water which, to a large extent, was directly used in photosynthesis. On the other hand, high Fe^3+^ levels hampered plant metabolism and increased phenolic compound production. The reduction in water uptake in this condition is due to the stress effects stimulated by iron at high concentrations. This limits the yield of cultivated herbs. In our studies, a decrease in the fresh mass of the above-ground parts of the plant was observed in all iron applications. We reason that the latter affects the plant growth strategy and prompts more energy to be invested into root system development. This hampers the growth of its green parts. Further studies are clearly needed for the determination of the Fe(NO_3_)_3_ levels which will elevate phenolic compound production in lemon balm while not substantially limiting plant metabolism.

## Figures and Tables

**Table 1:** Analysis of untreated soil.

pH	5.82			
Organic matter	25%			
Metal content (µg/g)	Fe^3+^	Mn^2+^	Cu^2+^	Zn^2+^
Total forms	3452 ± 11	168 ± 5	29.6 ± 0.6	94 ± 3
Bioavailable forms	442 ± 5	93.8 ± 1.1	18.5 ± 0.4	48.2 ± 0.9

**Table 2:** The one-way ANOVA for iron, manganese, copper and zinc concentrations in lemon balm plants across iron supplementation. Critical Snedecor’s F value is F_cryt_.(5,24) = 2.620; probability level *p* = 0.05.

	Mn^2+^	Cu^2+^	Zn^2+^
Roots	F = 343.636 *p* = 1.66 × 10^−21^	F = 295.300 *p* = 9.94 × 10^−21^	F = 432.119 *p* = 1.10 × 10^−22^
Above-ground parts	F = 393.066 *p* = 3.39 × 10^−22^	F = 166.168 *p* = 8.36 × 10^−18^	F = 132.868 *p* = 1.11 × 10^−16^

**Table 3:** Bioaccumulation factors (BAFs), translocation factors (TFs) and transfer coefficients (TCs) calculated for lemon balm. Elements are shown in decreasing order of particular factor. Each value is the average of the data from five replicates.

Samples	BAF	TF	TC
control	Mn(0.51) > Zn(0.36) = Cu(0.36) > Fe(0.03)	Mn(1.21) > Zn(0.62) > Cu(0.57) > Fe(0.26)	Cu(0.64) > Zn(0.58) > Mn(0.42) > Fe(0.12)
100 µg/g	Mn(0.50) > Zn(0.36) > Cu(0.31) > Fe(0.03)	Mn(1.24) > Zn(0.61) > Cu(0.56) > Fe(0.23)	Zn(0.59) > Cu(0.55) > Mn(0.41) > Fe(0.14)
200 µg/g	Mn(0.43) > Zn(0.32) = Cu(0.32) > Fe(0.04)	Mn(1.08) > Zn(0.59) > Cu(0.53) > Fe(0.28)	Cu(0.60) > Zn(0.55) > Mn(0.40) > Fe(0.15)
500 µg/g	Mn(0.40) > Cu(0.29) > Zn(0.20) > Fe(0.06)	Mn(1.26) > Cu(0.76) > Zn(0.48) > Fe(0.29)	Zn(0.41) > Cu(0.38) > Mn(0.31) > Fe(0.21)
700 µg/g	Mn(0.30) > Cu(0.22) > Zn(0.19) > Fe(0.09)	Mn(1.11) > Cu(0.77) > Zn(0.57) > Fe(0.37)	Zn(0.33) > Cu(0.28) > Mn(0.27) > Fe(0.23)
900 µg/g	Mn(0.26) > Zn(0.19) > Cu(0.14) > Fe(0.10)	Mn(1.44) > Cu(0.65) > Zn(0.59) > Fe(0.42)	Zn(0.31) > Fe(0.24) > Cu(0.22) > Mn(0.18)

**Table 4:** One-way ANOVA for index of chlorophyll, net photosynthesis, transpiration, intercellular concentration of CO_2_, stomatal conductance and dry mass of lemon balm across iron supplementation. Critical Snedecor’s F value is F_cryt_.(5,24) = 2.620; probability level *p* = 0.05.

Index of Chlorophyll	Net Photosynthesis	Transpiration	Intercellular Concentration of CO_2_	Stomatal Conductance	Dry Mass
F = 811.055 *p* = 6.20 × 10^−26^	F = 262.826 *p* = 3.90 × 10^−20^	F = 238.386 *p* = 1.20 × 10^−19^	F = 555.339 *p* = 5.62 × 10^−24^	F = 132.980 *p* = 1.10 × 10^−16^	F = 687.045 *p* = 4.50 × 10^−25^

**Table 5:** Conditions for measuring photosynthesis parameters.

Parameter		Value
Chamber CO_2_ concentration	CO_2r_	254.7 μmol·mol^−1^
Light intensity	PARi	1737 μmol m^−2^·s^−1^
Leaf temperature	Tleaf	28.9 °C
Vapor pressure deficit	VPD	2.2 kPa

## Data Availability

The original contributions presented in this study are included in the article and Supplementary Materials. Further inquiries can be directed to the corresponding author.

## Supplementary Materials

The following supporting information can be downloaded at: https://www.mdpi.com/article/10.3390/ijms27146211/s1.
