# Supplementary material for: Iron Nitrates Stimulate Phenolic Compound Production by Melissa officinalis L
Source: Int J Mol Sci. 2026 Jul 11;27(14):6211. doi: 10.3390/ijms27146211 (PMC13410267; doi:10.3390/ijms27146211)
Supplement: Supplementary file 1 [file ijms-27-06211-s001.zip › ijms-4347726-supplementary.pdf]

Supplementary materials

Table S1. Metals concentrations in the certified reference material ($\bar{x} \pm ts_{\bar{x}}$; p = 0.95, n = 5).

Metal	Certified value µg/g	Found µg/g	Recovery %
**Manganese**	191 ± 12	188 ± 7	98
**Copper**	7.77 ± 0.53	7.48 ± 0.43	96
**Zinc**	33.5 ± 2.1	33.1 ± 1.7	98
